# Comparative Analysis of the Nitrogen Effect of Common Agricultural Practices and Rotation Systems in a Rainfed Mediterranean Environment

**DOI:** 10.3390/plants6040061

**Published:** 2017-12-06

**Authors:** Panagiotis Dalias, Damianos Neocleous

**Affiliations:** Department of Natural Resources and Environment, Agricultural Research Institute, Nicosia 1516, Cyprus; d.neocleous@ari.gov.cy

**Keywords:** nitrogen effect, vetch-wheat rotation, fallow, sewage sludge application, straw incorporation, dry Mediterranean climate

## Abstract

The nitrogen (N) effect of legumes is one of the main reasons for their inclusion in rotation systems and their success in rainfed agriculture of Mediterranean areas. The comparative analysis of this effect in relation to alternative systems or practices is essential for a comprehensive appreciation in their merit. This field experiment was comprised of four three-year rotation cycles. Wheat (*Triticum turgidum durum*) was seeded for two consecutive years after common vetch (*Vicia sativa* L.), treated in three different ways, and after fallow and compared with three wheat monocultures: the conventional one, the continuous straw incorporation, and the sewage sludge incorporation once every three years. Wheat grain and straw yields and N uptake were compared among treatments. Results showed that rotation systems that included vetch were the most promising for improving sustainability. Maximum N uptake and the greatest yield surpluses were obtained when wheat followed vetch incorporated during flowering. When vetch in the rotation was cut for hay or left to fill grains subsequent wheat showed also enhanced yields. Fallow affected the rotation system’s fertility due to the incorporation of volunteer plants into the soil. Sewage sludge sustained production without the need for inorganic fertilization during three years. Straw incorporation always gave the smallest yields and N harvests, presumably due to soil N immobilization.

## 1. Introduction

Food security is a major concern for the Mediterranean region, due to ongoing climatic changes and to major losses of agricultural land to urban expansion, particularly along the coast, which is heavily influenced by tourism. Agronomic management choices to improve food production are in a close relationship with economic considerations, and, therefore, comparative studies of agricultural practices under field conditions could be a useful tool. Such approaches would indicate not only the direction of change, which to a great extend is already known, but provide results on the magnitude of the difference between these practices, and thus facilitate their financial appreciation.

Cereal mono-cropping systems are common in Mediterranean areas due to their simplicity, as there is focus on cultivation and management issues related to only one species. However, mono-cropping systems provide only temporary economic advantages and have significant agronomic and ecological drawbacks [1]. In the long term, they result in reduced soil quality, which is manifested by reduction in soil fertility, greater water and wind soil erosion rates, and damage of soil structure [2]. Continuously growing the same crop in a field will tend to exploit the same root zone, which can lead to a decrease in available nutrients for plant growth and a less effective exploitation of soil water [3]. Moreover, the worsening of most insect pest problems is increasingly linked to the expansion of crop monocultures [4].

As a response to soil deterioration problems and progressive loss of fertility in rainfed Mediterranean fields, a number of changes in agricultural practices have been proposed. They can be grouped in two broad categories: practices that aim at the increase or maintenance of soil organic matter [5] and the changeover from monoculture to crop rotations that may include a year of fallow [6].

Conservation tillage [7] and the incorporation in soil of crop residues, which are otherwise burned or removed for forage, are usually associated with a number of benefits. These include, apart from the increase in soil organic matter, the improvement of soil structure, the reduction of leached nitrate during the heavy rain periods, and the enhancement of soil microbial biomasses [8,9,10]. However, the reported effects of straw incorporation on crop yields in cereal-dominated cropping systems are inconsistent [11]. Sewage sludge, meeting regulatory criteria in terms of limits for heavy metals, pathogens, and nutrient loading, applied to agricultural soils can also enhance microbial activity and crop production [12].

Fallowing, the practice of leaving the soil unplanted for some time so as to collect and conserve more rain water for a subsequent crop, is particularly relevant to drought-prone areas where rainfall is often inadequate for consistently good crop yields. Evidently, in a Mediterranean type of climate or a shallow soil, where little water may sustain the long, hot, dry summers until the next crop is planted, fallow is not always an effective practice [13].

Most of cereal monoculture drawbacks are usually avoided by rotating crops in the same field, thus achieving a greater sustainability [14]. The inclusion of legumes in rotation systems in particular, represents one of the main modes for improving productivity in conventional rainfed agriculture and an even more central choice in organic farming.

Apart from the ‘break crop effect’, which is the reduction of the potential of pests, diseases, and weeds when sequences of similar crops are ‘broken’ by an alternative crop, legumes are widely appreciated for the provision of nitrogen to subsequent crops (‘nitrogen effect’), which, therefore, receive lower levels of fertilizer. Nitrogen supplied by legumes disperses throughout the soil profile, and is more effectively retrieved by wheat compared with surface applied N fertilizer [15]. If part of the soil mineral N that is provided to subsequent crops after the decomposition of legume residues has been gained through symbiotic N fixation, then the agricultural system is being indirectly enriched by atmospheric N [16,17].

The inclusion of common vetch, which fixes nitrogen without inoculation, has been proved to be the best legume to include in cereal-based rotations in relation to its effect on N nutrition, at least in Cyprus, as it delivers great quantities of mineralized N to the following crop [18,19,20]. Vetch is usually incorporated in soil during flowering. Harvesting vetch as forage or leaving it to fill grain are seemingly less productive practices in adding external N to the system because a greater amount of N is removed from the site; as hay or in the grain at harvest [21].

The aim of the study, which lasted eight years, was to quantify the yield and nitrogen benefits of vetch in three-year rotation systems in relation to wheat monoculture and evaluate from this respect three vetch management practices (vetch for hay, seed production, or soil incorporation). These effects were compared to those of fallow and to two practices of continuous organic matter addition; straw and dry sewage sludge. The comparative analysis of the “nitrogen effect” of practices and rotation systems is expected to provide useful information for the cost of the related agronomic choices and their associated fertilizer replacement values, but also for their environmental evaluation in dry Mediterranean climates.

## 2. Results

Wheat grain yields at the same plot varied greatly from one year to another. At the control cereal monoculture treatment (“cnvl”) for example, grain yield production in plots ranged from 1.4 to 5 t ha^−1^. Simple correlation analysis between these yields and annual rainfall, which to a great extent coincides with the rainfall of the growing season in Cyprus, did not show any statistical significance (results not shown). Similar analysis was also attempted using November–May or February–May precipitation data, but although Pearson’s *coefficient* was always positive, the correlation was not significant. The interannual variation and the lack of correlation with rainfall justified the analysis of treatment effects to be founded on results of the same year.

Results of the first year of each rotation cycle are presented separately from results of the second year, which are briefly denoted as “test year” results, and third year results, mentioned as “residual effect years”. First year results refer to yields and N characteristics of the three continuous wheat treatments only, whereas “test year” and “residual effect year” results to all of the treatments. Tables 1, 2 and 4 contain data on grain production, straw production, and N concentration in grain or straw, which represent primary data, derived directly from measurements, but contain also derived data: total biomass, which is the sum of grain and straw production, total N in grain or straw, which is the result of grain or straw production (on a dry basis this time) with N concentration in grain or straw, respectively, Nitrogen Harvest Index = total N in grain/total N in aboveground plant biomass (whole grain + straw) and Grain Harvest Index = grain production/aboveground plant biomass.

### 2.1. First Year Results

Table 1 on first year results of wheat shows also aboveground vetch production of the “vgrn” treatment and the concentration of N in vetch plant biomass. These data provide an indication of the amount of forage that was removed from “vhay” treatments and the amount and N content of residues that were incorporated in soil in “vinc”.

Plots that received sewage sludge at the beginning of the growing season showed greater production of wheat grain in relation to “cnvl” at only one of the four rotation cycles. The “sldg” treatments showed that the total amount of N that was contained in straw was greater than in “cnvl”, even in cases when straw yield or N concentration in straw biomass was not significantly different than “cnvl” (years 2007, 2010). Average grain or straw yield in “strw” ranked always last (Table 1).

### 2.2. “Test Years”

**Figure 1:**
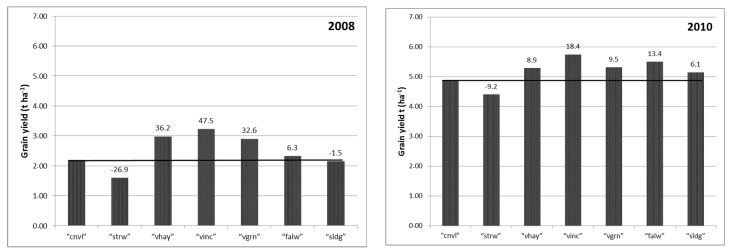

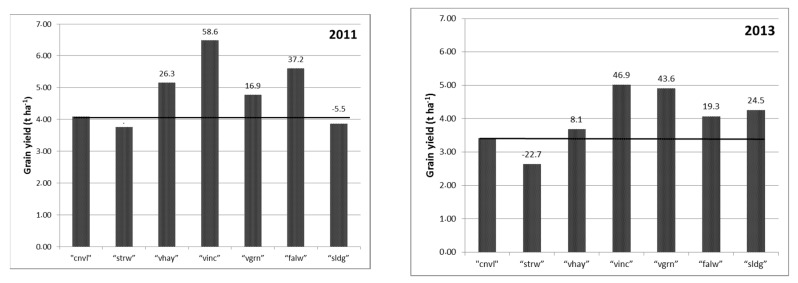
Average grain yield production in t ha^−1^ obtained at the “test years” of the four rotation cycles. Numbers above the columns indicate yield surpluses, i.e., the percentage difference of this production in relation to the “cnvl”.

In all four of the rotation cycles that were followed in the study, wheat grain production after vetch incorporation during flowering was the greatest and significantly different than “cnvl” (and “strw”) treatments (Table 2). The “vinc” plots yielded on average 42.9% more grain than the “cnvl” plots (Figure 1).

Average grain yield surpluses were also shown by “vgrn” treatments in relation to “cnvl” but in two out of four rotation cycles differences were not statistically significant. Similarly, “vhay”, “falw”, and “sldg” resulted in greater than “cnvl” mean grain yields, but these increases were statistically significant in only one rotation cycle (Table 2).

Much smaller differences between treatments were obtained for straw production. Overall, in only one of the four rotation cycles, “vinc” and “falw” showed greater wheat straw production than “cnvl”. The production of leaves and stems was significantly different in some rotation cycles between the least performing wheat in “strw” and the best performing “vinc” or “falw” (Table 2).

Nitrogen concentration in grain was found to be a much more “conservative” variable than grain yield in relation to the treatment effect. In only one cycle some statistically significant differences were found between treatments while in the other three cycles N concentration was not affected. N concentration in “vinc” was never significantly greater than “cnvl”. Dough quality characteristics, which are closely related to N concentration, but also to the “quality” of protein in the flour, are indicated by the results shown in Table 3. As with N concentration results differences in means between treatments were not always statistically significant. Dry gluten and alveograph W values shown by “vinc” were different from “strw” but not from “cnvl”. Farinograph water absorption results showed statistically significant differences between treatments even in cases where N concentration and gluten content did not vary.

However, the total amount of N that was accumulated in grain, which is a function of grain production and N concentration in grain tissues, was significantly greater in “vinc” than in conventionally cultivated monoculture plots.

Nitrogen harvest index and grain harvest index were used to indicate the physiological adaptations of crops to soil N availability, as they expressed by the allocation of N and biomass, respectively, to grain in relation to the whole aboveground plant. Treatments appeared to affect both of the indices. However, in treatments of enhanced wheat growth, crops allocated a greater proportion of their total biomass to grain even in cases where N allocation was not affected. At all of the rotation cycles, for example, “vinc” plots showed greater grain harvest indices than “cnvl” plots during the test years, whereas for the same plot comparison, N harvest index was greater in only one of four rotation cycles (Table 2).

### 2.3. “Residual Effect Years”

Third year results at each cycle revealed residual effects of vetch cultivation and fallow that were implemented during the first year of the rotation.

Fallow was the only treatment that gave grain surpluses in relation to “cnvl” at all of the cycles, although differences were not always statistically significant. Moreover, in one cycle the total amount of N that was accumulated in grains at the “falw” plots was significantly greater than at the “cnvl” plots. This effect was not observed during the “test years” (Table 4). During the “residual effect years” of the rotations, vetch treatments did not show the greatest grain productions and differences between these and the “cnvl” were not consistent (Table 4). “Vinc” for example showed greater average yield in relation to “cnvl” in 2009, but lower in 2012. Significantly lower grain productions were shown by “vhay” and “vgrn” treatments in 2011 and 2012, but in all other cases, vetch treatments, which did not receive any fertilization for second consecutive year, sustained production equivalent to “cnvl” (Table 4).

At the third growing season after sludge application, “sldg” treatments were also not significantly different from “cnvl” in relation to grain or straw production.

For two of the four rotation cycles the total production of grain in “vinc” during the test and residual years was significantly greater than in “cnvl”, while “vgrn” and “falw” had also one cycle with greater than “cnvl” cumulative production during these years.

## 3. Discussion

**Figure 2:**
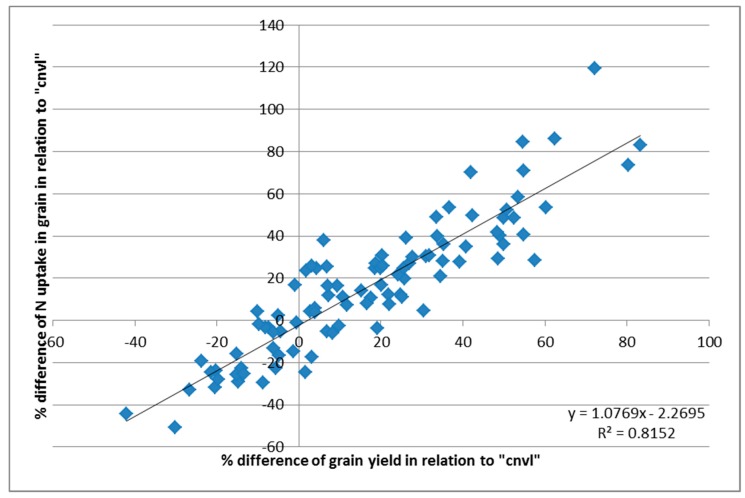
Correlation between the percentage difference of grain yield in relation to “cnvl” and the percentage difference of the total N uptake by grain during the “test years” of the experiment. Paired data were calculated from results that were obtained inside the same block (see Figure 3).

The percentage change of grain yield in relation to “cnvl” during the “test years” of the experiment correlated very well with the corresponding percentage change of the total N uptake by grain tissues. This is illustrated in Figure 2. The straight line that was fitted to data passes very close to the origin and slope, and was very close to 1, indicating that any change in grain yield was closely associated to N availability. It could be reasonably stated that N was the cause of any grain production increase in this experiment, or at least that, if other factors are responsible for such an increase, this would be realized only if it is “supported” by soil N availability.

Vetch residues remaining or returned to soil are the main source of available N supply to the following cereal crop at the vetch-wheat rotation systems. The greatest positive effects of vetch were manifested when shoot residues were incorporated in soil during flowering, justifying, to a great extent, the use of this rotation system in Cyprus and other dry Mediterranean regions as the main system for improving soil fertility [14,18].

Results clearly showed that between the three vetch systems that were studied, “vhay” was the worst in relation to its effect on the subsequent wheat. This is presumably due to the smaller quantity and lower quality of residues (only roots) that remained in the soil. However, these residues were sufficient to provide, together possibly with enhanced soil native organic matter decomposition, the extra N that was needed to support following year’s wheat production and a significant grain yield surplus. The possibility to cut vetch for forage is, therefore, a considerable option for farmers in dry areas, where there is a significant demand for hay and where rainfall in late stages of the growing season is often low, making the completion of the biological cycle of vetch uncertain. Nevertheless, since residual effects during the second year after incorporation were found to be small, vetch for hay seems a more suitable choice in two-year rotation systems.

The practice of leaving vetch to fill grains, which are harvested, removes from the field a great part of fixed N [21], and reduces the total amount of N that is incorporated in soil [22]. Part of N that is contained in leaves is translocated to grain as it was reported by [23,24] who noticed that crude protein contents in legumes decline as plants mature from the vegetative stage through the reproductive stages, particularly after flowering. This again did not nullify the positive effect of vetch to subsequent crops in the present experiment (see also [20]).

It is, therefore, clearly inferred that the inclusion in a rotation system of pulses or a legume that is cut for hay could have significant overall benefits [25], as it provides a more sustainable cropping system than cereal monoculture [26] and increases the producer’s income without compromising forage production and food security demand by providing a cheap source of protein for human and animals. It is emphasized that legume biomass incorporation in soil may be the optimum practice from the point of view of “N effect” but has the shortcoming to restrict the income of a producer in a three-year rotation system to only two years.

Lowest vetch incorporation effects (18.4% grain yield surplus) were obtained when the legume year was followed by a year with higher precipitation, whereas in the other three rotation cycles, when 1st years were followed by more dry “test years”, surpluses were much greater (47.5, 58.6, and 46.9%). Limiting rainfall conditions were expected to mask soil N availability effects [6,27], or at least influence them proportionally, but in the present results, greater percentage increases were shown in drier test years. It has been shown that vetch, but also food legumes like lentil and chickpea, have shallow roots and do not deplete soil moisture to the same extent as cereals, leaving some residual soil moisture for the succeeding crop [28,29,30,31]. Proportionally greater yields in “vinc” plots of this experiment could accordingly be explained by the synergistic effect of available moisture stored in the soil and N fertilization from legume residues [32]. Moreover, it has been shown that nutrient availability is important, even in low rainfall areas, as it maximizes water use efficiency [33,34].

High soil N fertility might result in a decrease in the deposition of water-soluble carbohydrates in wheat stems, tillers, and leaves, and an increase in structural carbon, leading to less mobilization of vegetative C to grain and a lowering of harvest index [35,36,37]. From this point of view, vetch showed in this experiment a different pattern of N provision to the subsequent crop. The decomposition of vetch residues increased both grain yield and grain harvest index, presumably due to a more even supply of N during the growing season, thus offering a greater production security.

The anticipated immobilization of N from the integration of plant residues with a large C/N ratio [38,39] can be reasonably considered as the cause of the generally smaller grain and straw yields and N uptake in “strw” treatments (Table 1, Table 2 and Table 4). These results emphasize the view that an eventual positive effect of incorporation of straw to soil fertility cannot become immediately evident in the climatic conditions of Cyprus. The duration of the favorable period for soil microbial activity is apparently not sufficient to allow the shift from net immobilization to net mineralization [40]. The expected increase in soil organic matter, soil microbial biomass, and improvement in soil infiltration and soil structure will probably be manifested in soil fertility characteristics after many years of continuous straw incorporation that overpass the duration of the current experiment [41,42].

Regarding the residual effects of treatments, dry sewage sludge application seemed to have a more lasting influence on soil mineral N provision in relation to vetch. For three consecutive growing seasons, sludge sustained crop production without inorganic fertilization (Table 1, Table 2 and Table 4). The average sum of grain production for all three years of the rotation was 12.09 t ha^−1^ for “sldg”, in comparison to 11.77 for “cnvl” and 9.89 t ha^−1^ for “strw”.

Conservation of soil water during fallow has been called upon as an explanation of increased yields after fallow [43]. Under the climatic conditions of Cyprus, fallow did not always result in water storage in soil and this water had not always a positive effect to the subsequent cereal [44]. Carry over soil moisture should be expected to be greater in nutrient poor soils. Rich soils, as the one that current experiment took place, develop wild vegetation that respires a greater part of water, which would become available to the subsequent crop.

However, one of the most surprising results in the present experiment was the production and ranking of “falw” in relation to other treatments two years after fallowing. Fallow plots that received the same fertilization as conventional plots produced more grain in years when no conserved soil water effect is expected. The incorporation of wild vegetation during fallowing is a form of green manuring with, nevertheless, a more slowly decomposing plant biomass in relation to vetch. Nitrogen effects of wild plant residues on crop production seemed to be allocated almost equally to the two years following fallow, and were made more evident the second growing season after incorporation. More precisely, the mean total N in harvested biomass of “cnvl” was 94.33 kg ha^−1^ during the test years and 97.62 kg ha^−1^ during the residual years. The easily decomposable vetch residues with high N concentration increased wheat harvested N in “vinc” test years to 126.84 kg ha^−1^ without inorganic fertilization, but this rapidly dropped to 92.28 kg ha^−1^ during the “residual years”. On the contrary, the slower decomposing wild vegetation in “falw” resulted in a total accumulation of 105.05 kg ha^−1^ N at the “test years”, and 106.91 kg ha^−1^ N at the “residual years”.

Results on the effect of rotation, fallow and sludge application should be appreciated not only in relation to grain or straw yield surpluses but also in terms of the quantity and quality of the contained protein and the dough quality indices that are associated with it. As it was shown by the results of 2008 when some grain quality characteristics were estimated, better soil N nutrition of wheat positively affected parameters like gluten content and bread making characteristics of flour [45].

The acquired data on the N effect of agricultural systems and practices have significant repercussions on the viability of farming operations. The use of legume crops in rotations, for example, can reduce the need for additional synthetic nitrogen fertilizer, lower costs, and increase profit margins. Present data, however, also provide indications on the environmental impact of the studied agronomic choices. Reduced synthetic fertilizer use in legume rotations leads to reduced greenhouse gas emissions from the manufacturing process and transportation [46]. When nitrogen is provided to plants by organic sources, such as legume residues in rotation systems, nitrate leaching or runoff is reduced [47]. In these systems, soil biodiversity rises [48], but cereal nematode populations are reduced, so as the need for soil nematocide treatments [49]. A thorough evaluation of practices, therefore, needs to take into account not only economic aspects, such as yield surpluses, greater quality due to higher protein, gains from grain or forage sale, but also environmental benefits.

## 4. Materials and Methods

The experiment was carried out in an experimental station of the Agricultural Research Institute (ARI) of Cyprus (34°44′ Ν; 32°29′ Ε; elevation, 30 m) near the city of Paphos. Soil in the study site is characterized as clay loam (Vertic Luvisol), with CaCO_3_ content of 15%, pH of 7.9 and organic matter content of 1.5% (Table 5).

**Figure 3:**
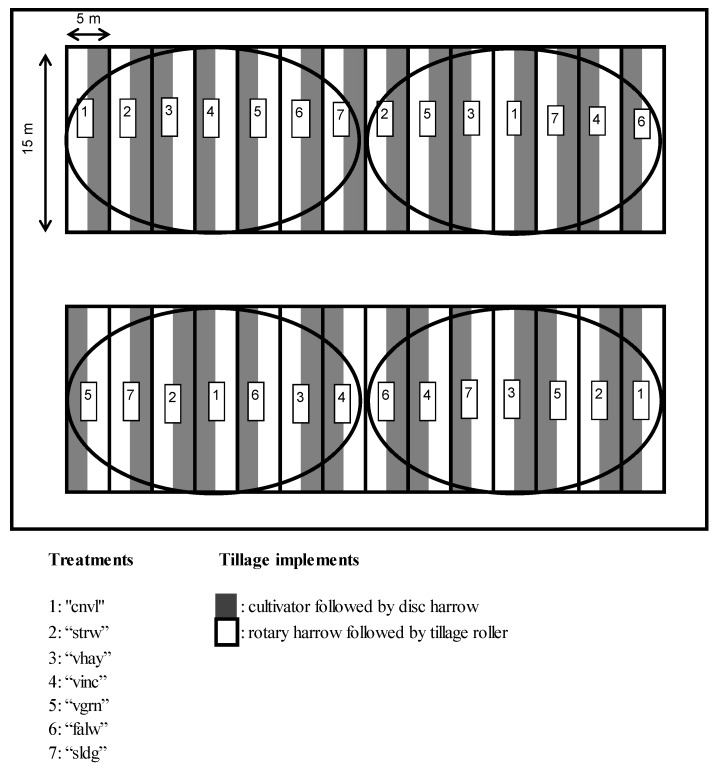
Drawing showing the arrangement of plots in each of the two adjacent fields where the rotation experiment took place. Numbers 1–7 indicate the seven treatments, which were the main plots, and which were grouped in four blocks. The explanation of treatment abbreviations is given in the text.

The experimental design was a split-plot with four replications (randomized block), and is shown in Figure 3. Each block had an area of 35 m × 15 m = 525 m^2^ and the experiment in each field extended, including corridors, in a total area of about 0.6 ha. The main plots were the different agricultural practices or agricultural systems and the subplots were two tillage treatments, in a randomized arrangement. The subplot size was 2.5 m × 15 m = 37.5 m^2^ and the main plot size was 5 m × 15 m = 75 m^2^. The subplots were strips of land cultivated with two different tillage machines: (1) cultivator followed by disc harrow, and (2) rotary harrow followed by tillage roller. Results and discussion below refer only to the comparison between agricultural practices and systems (main plots in the experimental design), and not to the effect of tillage (sub-plots), which is given no further analysis. This analysis showed that there was no interaction between tillage systems and the main plot treatments so all calculations and statistical analyses were carried out using means of the two tillage treatments for each main plot to make results more robust.

The same management practices, rotation systems, and experimental design, as shown in Figure 3, were applied in two adjacent fields of the same soil type. Cultivation at the second field started two years after the first to provide variation of the weather conditions among the three-year rotation cycles. The experiment lasted eight years and comprised four three-year cycles. Legume, fallow and sludge application were carried out during the first year of each cycle. Test crop yields, i.e., wheat yields at the second year of each cycle, were thus obtained in 2008 and 2011 in one field and in 2010 and 2013 at the other. Residual effects at each treatment were revealed by results of the third year of each cycle.

The main treatment characteristics are given below, together with the abbreviations with which they are quoted in the rest of the text. In three of the seven treatments, plots were seeded with durum wheat (*Triticum turgidum durum* var. Hekabe—purified variety enlisted in the national catalogue of varieties of Cyprus) during three consecutive years (continuous wheat cultivations), in three others common vetch (*Vicia sativa* L. var. Kimon—variety enlisted in the national catalogue) was seeded at the first year followed by wheat during the next two years, and in the last one, plots were left fallow the first year followed by two years of wheat.

The seven treatments of the experiment were as follows:

1. Conventional treatment (abv. “cnvl”). Continuous wheat system, where plots were fertilized at a rate of 300 kg ha^−1^ year^−1^ of a 20-20-0 fertilizer as pre-sowing fertilization and 90 kg ha^−1^ year^−1^ of a 34.5-0-0 fertilizer as top dressing. The total amount of N added was 90 kg ha^−1^. These fertilization practices are commonly used in many areas of Cyprus and do not vary according to the soil nutritional status, which is generally considered to be low. Straw was removed from the plots after harvesting.
2. Straw incorporation (abv. “strw”) in a continuous wheat system. Straw in Cyprus is most frequently baled after harvesting, removed from the field and used as forage. In this treatment straw was incorporated in soil at a rate of 5.3 t ha^−1^ for the first year. From the second year onwards, the amount of straw added was that harvested in the plots of this treatment, after its temporary removal by a threshing machine for weighing. Inorganic fertilizers were added as in “cnvl”.
3. Vetch for hay (abv. “vhay”). Soil was seeded with vetch the first year, received 100 kg ha^−1^ of a 0-48-0 phosphorus fertilizer only for that year, and left without inorganic fertilization for the following years of wheat crop. Aboveground biomass of vetch was cut during early stages of flowering and was removed from experimental plots.
4. Vetch incorporation (abv. “vinc”). Plots were seeded with vetch the first year and treated as in “vhay” with no nitrogen fertilization, but aboveground biomass was incorporated in soil during flowering using rotavator.
5. Vetch for seed (abv. “vgrn”). Plots were seeded with vetch the first year and treated as in “vhay” and “vinc”, but vetch was left to seed. After seed harvesting aboveground biomass was incorporated in soil.
6. Fallow treatment (abv. “falw”). Plots were left fallow during first year followed by wheat the second and third year. Wild vegetation was mechanically destroyed at the beginning and the middle of the growing season. Wheat at the second and third year of the rotation was fertilized as in “cnvl” and “strw”.
7. Sewage sludge application (abv. “sldg”) in a continuous wheat system. Sludge, some characteristics of which are shown in Table 5, had been air dried in the municipal waste water treatment plant for more than six months after anaerobic digestion. It was added to soil only the first year of each rotation cycle at a rate of 15 t ha^−1^ (dry weight basis). Plots that received sludge were not given any other fertilizer during the whole period of the rotation.

Seeding was carried out at the end of November or at the beginning of December, depending on soil moisture conditions. Soil properties that are shown in Table 5 were taken one week before seeding at the beginning of the experiment in 2006. Soil samples were taken from the surface (0–10 cm depth) after removing by hand visible plant residues and roots. They were then air dried and sieved with a 2 mm sieve. Harvesting was carried out in the middle of each of the 2.5 m wide sub-plot lines using a small combine harvester with a 1.5 m wide header. After harvesting, grain and straw yield was weighted and their N contents were measured (Kjeldahl N).

Samples of seeds harvested in 2008 were ground and flour content in gluten was measured with Glutomatic 2200 (Perten Instruments, Hamburg, Germany). Their alveograms (Model Alveographe NG, Chopin, France) and farinograms (GmbH Co., Brabender and Duisburg, Germany) were also used to provide insights on the differences in dough characteristics between treatments. From the various specific properties that are measured in a farinograph test of flour the water absorption is presented below as a resultant of gluten quantity and quality, while from alveograph results, the *W* value was chosen to indicate the baking strength of dough.

### 4.1. Meteorological Data

**Figure 4:**
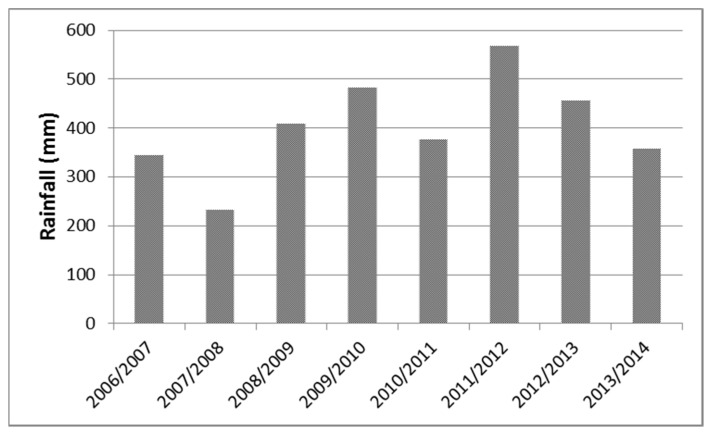
Cropping season (September to May) rainfall obtained from the closest meteorological station for each year of the experimental period.

Meteorological data taken from the closest weather station are shown in Figure 4. Fluctuations over the years largely represent variations of mean annual rainfall over the whole Cyprus. Average annual rainfall in August 2007 in Cyprus was 272.3 mm or 54% of the pluriannual mean, which is estimated by the World Meteorological Organization for the years 1961–1990. This rainfall was the second lowest since the beginning of the previous century and it was not sufficient to support production of cereals in most other districts of Cyprus apart from Paphos, where the experiment took place. The hydrometeorological year 2011/2012 was the wettest of the eight years of the experiment both in the whole Cyprus (655 mm) and in the experimental site (568 mm).

### 4.2. Statistical Analysis

Comparisons between treatments on a given variable at the same year were carried out using ANOVA and Tukey as posttest (Prism, Graphpad Software, Inc., San Diego, CA, USA). Measurements at the same block (Figure 3) were considered as matched subjects. In Prism, the statistical analysis test used for such designs is called “repeated measures ANOVA” and is the same for both repeated measures and randomized block experiments as in the present study (in Microsoft Excel the respective test is called “two-way ANOVA without replication”). The interaction of rotation cycle with treatment effects was tested using two-way ANOVA and Bonferroni as posttest, always keeping matching between subjects of the same block in the field (Prism, Graphpad Software, Inc., San Diego, CA, USA).

## 5. Conclusions

Efficient management practices could substantially lower nitrogen use as fertilizer or increase nitrogen/protein in plant tissues. The comparison of biomass and N yields between common management practices and agricultural systems that was attempted in this study clearly showed that simple changes of rotation system or soil amendment could have a significant impact on the income of producers and food production.

Breaking wheat monoculture with vetch, which was incorporated in soil or cut for hay or left to fill grains, increased the yields of the following wheat marking these systems as the most promising for improving sustainability. Fallow affected also system’s fertility status, due to the incorporation in soil of the wild vegetation. Sewage sludge sustained production without inorganic fertilization during three years. Results provide data for evaluating the agronomic and environmental cost of the replacement of continuous wheat systems.

## Figures and Tables

**Table 1:** Mean wheat yields and N concentration and uptake at the first year of each rotation cycle. Statistical analysis results displayed at the right side of values refer to the comparison between treatments and the “cnvl”. Results in the “vgrn” column refer to vetch.

Rotation Cycle No./Year of Harvesting	“cnvl”	“strw”	“vgrn”	“sldg”
**Grain yield (t ha^−1^)**				
**1/2007**	5.60	4.02 *		6.03 n.s.
**2/2009**	4.82	4.22 n.s.		4.27 n.s.
**3/2010**	3.83	3.68 n.s.		3.46 n.s.
**4/2012**	4.50	3.81 n.s.		6.08 **
**Straw yield (t ha^−1^)**				
**1/2007**	6.71	5.51 *	4.50	7.83 n.s.
**2/2009**	5.90	4.80 n.s.	4.96	5.74 n.s.
**3/2010**	5.81	5.07 n.s.	4.40	4.61 n.s.
**4/2012**	5.79	4.75 n.s.	4.23	8.18 **
**Aboveground biomass (t ha^−1^)**				
**1/2007**	12.31	9.53 *		13.86 n.s.
**2/2009**	10.72	9.02 n.s.		10.01 n.s.
**3/2010**	9.64	8.75 n.s.		8.07 n.s.
**4/2012**	10.29	8.56 *		14.26 ***
**N concentration in grain (%)**				
**1/2007**	2.21	2.05 n.s.		2.32 n.s.
**2/2009**	2.25	2.33 n.s.		2.49 *
**3/2010**	2.09	1.89 *		1.95 n.s.
**4/2012**	2.50	2.29 n.s.		2.57 n.s.
**N concentration in straw (%)**				
**1/2007**	0.39	0.40 n.s.	1.69	0.49 n.s.
**2/2009**	0.44	0.45 n.s.	1.57	0.53 n.s.
**3/2010**	0.47	0.36 n.s.	1.63	0.37 n.s.
**4/2012**	0.64	0.70 n.s.	1.42	0.69 n.s
**Total N in harvested grain (kg ha^−1^)**				
**1/2007**	111.99	74.23 **		127.01 n.s.
**2/2009**	97.81	88.37 n.s.		83.21 n.s.
**3/2010**	71.12	62.18 n.s.		59.60 n.s.
**4/2012**	99.96	77.69 n.s.		138.69 *
**Total N in harvested straw (kg ha^−1^)**				
**1/2007**	24.34	20.69 n.s.		35.69 *
**2/2009**	24.70	20.13 n.s.		27.26 n.s.
**3/2010**	25.77	16.77 *		15.92 *
**4/2012**	33.84	30.23 n.s.		50.86 **
**Total N in aboveground biomass (kg ha^−1^)**				
**1/2007**	136.33	94.92 *		162.70 n.s.
**2/2009**	122.50	108.50 n.s.		110.47 n.s.
**3/2010**	96.88	78.95 n.s.		75.52 *
**4/2012**	133.80	107.92 *		189.56 ***
**Harvest index**				
**1/2007**	0.4549	0.4180 *		0.4344 n.s.
**2/2009**	0.4498	0.4684 n.s.		0.4357 n.s.
**3/2010**	0.3970	0.4253 n.s.		0.4291 n.s.
**4/2012**	0.4381	0.4439 n.s.		0.4264 n.s.
**N harvest index**				
**1/2007**	0.8213	0.7797 n.s.		0.7800 n.s.
**2/2009**	0.8016	0.8146 n.s.		0.7531 n.s.
**3/2010**	0.7383	0.7883 n.s.		0.7902 n.s.
**4/2012**	0.7472	0.7190 n.s.		0.7316 n.s.

* *p* < 0.05, ** *p* < 0.01, *** *p* < 0.001, n.s.: not significant.

**Table 2:** Treatment effects on wheat yields and N concentration and uptake at the second year of each rotation cycle (“test years”). Statistical analysis results displayed at the right side of values refer to the comparison between treatments and the “cnvl”.

Rotation Cycle No./Year of Harvesting	“cnvl”	“strw”	“vhay”	“vinc”	“vgrn”	“falw”	“sldg”
**Grain yield (t ha^−1^)**							
**1/2008**	2.19	1.60 n.s.	2.99 *	3.24 **	2.91 *	2.33 n.s.	2.16 n.s.
**2/2010**	4.85	4.41 n.s.	5.28 n.s.	5.74 **	5.31 n.s.	5.50 n.s.	5.14 n.s.
**3/2011**	4.09	3.76 n.s.	5.16 n.s.	6.48 ***	4.78 n.s.	5.61 *	3.86 n.s.
**4/2013**	3.41	2.64 n.s.	3.69 n.s.	5.01 ***	4.90 ***	4.07 n.s.	4.25 *
**Straw yield (t ha^−1^)**							
**1/2008**	3.88	3.42 n.s.	3.99 n.s.	4.67 n.s.	4.12 n.s.	4.13 n.s.	3.54 n.s.
**2/2010**	7.07	6.38 n.s.	6.90 n.s.	7.12 n.s.	7.03 n.s.	7.29 n.s.	6.70 n.s.
**3/2011**	5.59	5.37 n.s.	5.69 n.s.	6.94 *	6.16 n.s.	6.89 *	5.33 n.s.
**4/2013**	4.62	3.82 n.s.	4.31 n.s.	5.29 n.s.	5.61 n.s.	5.20 n.s.	4.78 n.s.
**Above ground biomass (t ha^−1^)**							
**1/2008**	6.07	5.02 n.s.	6.98 n.s.	7.90 **	7.03 n.s.	6.46 n.s.	5.70 n.s.
**2/2010**	11.92	10.78 n.s.	12.18 n.s.	12.87 n.s.	12.34 n.s.	12.79 n.s.	11.84 n.s.
**3/2011**	9.68	9.13 n.s.	10.85 n.s.	13.43 **	10.94 n.s.	12.50 *	9.19 n.s.
**4/2013**	8.04	6.46 n.s.	7.99 n.s.	10.30 *	10.51 *	9.27 n.s.	9.03 n.s.
**N concentration in grain (%)**							
**1/2008**	2.12	1.77 n.s.	2.01 n.s.	2.48 n.s.	2.09 n.s.	2.05 n.s.	1.94 n.s.
**2/2010**	1.95	1.97 n.s.	2.03 n.s.	2.06 n.s.	2.03 n.s.	2.00 n.s.	2.05 n.s.
**3/2011**	2.19	2.16 n.s.	2.15 n.s.	2.18 n.s.	2.20 n.s.	2.08 n.s.	2.16 n.s.
**4/2013**	2.15	2.05 n.s.	1.96 n.s.	2.04 n.s.	2.06 n.s.	1.87 n.s.	2.14 n.s.
**N concentration in straw (%)**							
**1/2008**	0.51	0.35 n.s.	0.40 n.s.	0.51 n.s.	0.42 n.s.	0.46 n.s.	0.36 n.s.
**2/2010**	0.46	0.47 n.s.	0.40 n.s.	0.45 n.s.	0.46 n.s.	0.43 n.s.	0.50 n.s.
**3/2011**	0.73	0.70 n.s.	0.60 n.s.	0.66 n.s.	0.69 n.s.	0.65 n.s.	0.69 n.s.
**4/2013**	0.43	0.40 n.s.	0.36 n.s.	0.36 n.s.	0.40 n.s.	0.37 n.s.	0.43 n.s.
**Total N in harvested grain (kg ha^−1^)**							
**1/2008**	41.59	24.78 n.s.	54.06 n.s.	70.23 ***	54.81 n.s.	41.89 n.s.	38.00 n.s.
**2/2010**	84.19	77.23 n.s.	95.22 n.s.	105.34 **	96.41 n.s.	97.71 n.s.	94.10 n.s.
**3/2011**	80.45	73.81 n.s.	100.27 n.s.	126.74 **	93.96 n.s.	104.13 n.s.	74.92 n.s.
**4/2013**	66.29	48.67 n.s.	65.57 n.s.	92.63 *	91.37 *	68.95 n.s.	82.00 n.s.
**Total N in harvested straw (kg ha^−1^)**							
**1/2008**	18.24	11.04 n.s.	14.49 n.s.	21.86 n.s.	16.03 n.s.	17.72 n.s.	11.91 n.s.
**2/2010**	30.51	28.13 n.s.	26.23 n.s.	29.99 n.s.	30.50 n.s.	29.59 n.s.	31.15 n.s.
**3/2011**	37.82	35.13 n.s.	31.89 n.s.	42.39 n.s.	40.09 n.s.	42.24 n.s.	34.73 n.s.
**4/2013**	18.26	14.40 n.s.	14.41 n.s.	18.18 n.s.	21.19 n.s.	17.96 n.s.	19.59 n.s.
**Harvest index**							
**1/2008**	0.3548	0.3155 n.s.	0.4324 **	0.4065 n.s.	0.4080 *	0.3592 n.s.	0.3754 n.s.
**2/2010**	0.4069	0.4083 n.s.	0.4347 n.s.	0.4467 *	0.4303 n.s.	0.4302 n.s.	0.4346 n.s.
**3/2011**	0.4227	0.4113 n.s.	0.4737 *	0.4827 *	0.4325 n.s.	0.4485 n.s.	0.4201 n.s.
**4/2013**	0.4273	0.4098 n.s.	0.4608 n.s.	0.4882 *	0.4668 n.s.	0.4398 n.s.	0.4724 n.s.
**N harvest index**							
**1/2008**	0.6877	0.6921 n.s.	0.7901 n.s.	0.7623 n.s.	0.7671 n.s.	0.7040 n.s.	0.7554 n.s.
**2/2010**	0.7359	0.7327 n.s.	0.7833 n.s.	0.7799 n.s.	0.7618 n.s.	0.7670 n.s.	0.7523 n.s.
**3/2011**	0.6795	0.6756 n.s.	0.7550 *	0.7498 *	0.6986 n.s.	0.7123 n.s.	0.6868 n.s.
**4/2013**	0.7839	0.7729 n.s.	0.8180 n.s.	0.8377 n.s.	0.8116 n.s.	0.7908 n.s.	0.8094 n.s.

* *p* < 0.05, ** *p* < 0.01, *** *p* < 0.001, n.s.: not significant.

**Table 3:** Summary of results of quality characteristics of flour coming from wheat grain harvested in 2008. Mean values followed by the same letter were not significantly different at *p* < 0.05.

	“cnvl”	“strw”	“vhay”	“vinc”	“vgrn”	“falw”	“sldg”
**Dry gluten (%)**	9.5 ab	7.4 a	8.9 a	12.0 b	10.0 ab	9.30 ab	8.6 a
**Farinograph water absorption (%)**	63.7 bcde	58.4 a	62.5 ad	67.6 b	64.2 bcde	62.7 ae	61.6 ac
**Alveograph W**	194.1 ab	130.6 a	154.3 ab	243.9 b	174.6 ab	150.3 a	155.1 ab

**Table 4:** Treatment effects on wheat yields and N concentration and uptake at the third year of each rotation cycle (“residual effect years”). Statistical analysis results appearing at the right side of values refer to the comparison between treatments and the “cnvl”.

Rotation Cycle No./Year of Harvesting	“cnvl”	“strw”	“vhay”	“vinc”	“vgrn”	“falw”	“sldg”
**Grain yield (t ha^−1^)**							
**1/2009**	2.74	2.07 n.s.	3.44 n.s.	3.93 n.s.	2.31 n.s.	4.48 *	2.70 n.s.
**2/2011**	3.90	3.23 **	3.15 **	3.77 n.s.	3.16 **	4.21 n.s.	3.50 n.s.
**3/2012**	4.35	3.70 n.s.	2.26 **	3.14 n.s.	2.87 *	4.51 n.s.	4.08 n.s.
**4/2014**	2.78	2.41 n.s.	2.20 n.s.	2.94 n.s.	2.81 n.s.	3.11 n.s.	2.82 n.s.
**Straw yield (t ha^−1^)**							
**1/2009**	6.09	7.25 n.s.	5.01 n.s.	5.41 n.s.	5.71 n.s.	7.19 n.s.	6.15 n.s.
**2/2011**	5.13	4.42 n.s.	4.53 n.s.	5.34 n.s.	4.48 n.s.	6.01 n.s.	4.67 n.s.
**3/2012**	4.84	4.31 n.s.	2.73 **	3.68 n.s.	3.29 *	4.73 n.s.	5.02 n.s.
**4/2014**	4.77	4.37 n.s.	3.68 n.s.	4.21 n.s.	4.04 n.s.	5.64 n.s.	4.81 n.s.
**Above ground biomass (t ha^−1^)**							
**1/ 2009**	8.84	9.32 n.s.	8.45 n.s.	9.34 n.s.	8.02 n.s.	11.67 n.s.	8.85 n.s.
**2/2011**	9.03	7.65 n.s.	7.68 n.s.	9.11 n.s.	7.64 n.s.	10.22 n.s.	8.17 n.s.
**3/2012**	9.19	8.01 n.s.	5.00 **	6.81 n.s.	6.16 *	9.24 n.s.	9.10 n.s.
**4/2014**	7.55	6.78 n.s.	5.88 *	7.15 n.s.	6.85 n.s.	8.74 n.s.	7.62 n.s.
**N concentration in grain (%)**							
**1/2009**	2.12	2.04 n.s.	2.20 n.s.	2.20 n.s.	2.17 n.s.	2.07 n.s.	2.18 n.s.
**2/2011**	2.24	2.17 n.s.	2.22 n.s.	2.22 n.s.	2.18 n.s.	2.24 n.s.	2.24 n.s.
**3/2012**	2.46	2.25 n.s.	2.05 ***	2.23 *	2.12 **	2.38 n.s.	2.18 **
**4/2014**	2.32	2.24 n.s.	1.69 ***	1.76 **	1.76 **	2.31 n.s.	2.08 n.s.
**N concentration in straw (%)**							
**1/2009**	0.55	0.54 n.s.	0.42 n.s.	0.35 n.s.	0.45 n.s.	0.34 n.s.	0.51 n.s.
**2/2011**	0.70	0.67 n.s.	0.62 n.s.	0.62 n.s.	0.63 n.s.	0.60 n.s.	0.64 n.s.
**3/2012**	0.60	0.60 n.s.	0.54 n.s.	0.60 n.s.	0.55 n.s.	0.57 n.s.	0.56 n.s.
**4/2014**	0.44	0.41 n.s.	0.29 *	0.31 n.s.	0.25 **	0.37 n.s.	0.36 n.s.
**Total N in harvested grain (kg ha^−1^)**							
**1/ 2009**	53.09	39.50 n.s.	69.76 n.s.	78.86 n.s.	45.38 n.s.	86.16 *	58.41 n.s.
**2/2011**	78.09	62.77 **	62.56 **	74.54 n.s.	61.94 **	84.11 n.s.	69.98 n.s.
**3/2012**	95.36	74.16 n.s.	41.43 ***	62.85 *	54.42 **	95.21 n.s.	78.13 n.s.
**4/2014**	71.24	53.19 n.s.	55.67 n.s.	79.06 n.s.	77.05 n.s.	84.54 n.s.	79.40 n.s.
**Total N in harvested straw (kg ha^−1^)**							
**1/2009**	13.22	17.19 n.s.	8.03 n.s.	8.01 n.s.	10.07 n.s.	10.03 n.s.	12.61 n.s.
**2/2011**	34.49	28.10 n.s.	27.19 n.s.	31.12 n.s.	26.40 n.s.	24.92 n.s.	27.66 n.s.
**3/2012**	26.22	23.28 n.s.	13.22 **	19.39 n.s.	16.36 *	24.46 n.s.	25.32 n.s.
**4/2014**	18.76	14.12 n.s.	11.52 n.s.	15.32 n.s.	13.04 n.s.	18.24 n.s.	16.10 n.s.
**Harvest index**							
**1/2009**	0.3163	0.2267 n.s.	0.4021 n.s.	0.4290 **	0.2967 n.s.	0.3818 n.s.	0.3135 n.s.
**2/2011**	0.4357	0.4301 n.s.	0.4084 n.s.	0.4148 n.s.	0.4130 n.s.	0.4116 n.s.	0.4302 n.s.
**3/2012**	0.4747	0.4646 n.s.	0.4530 n.s.	0.4598 n.s.	0.4658 n.s.	0.4906 n.s.	0.4453 n.s.
**4/2014**	0.3699	0.3548 n.s.	0.3758 n.s.	0.4100 n.s.	0.4096 n.s.	0.3565 n.s.	0.3696 n.s.
**N harvest index**							
**1/2009**	0.7911	0.6918 n.s.	0.8796 n.s.	0.9100 n.s.	0.8141 n.s.	0.8901 n.s.	0.8127 n.s.
**2/2011**	0.7006	0.6972 n.s.	0.7001 n.s.	0.7067 n.s.	0.7014 n.s.	0.7066 n.s.	0.7163 n.s.
**3/2012**	0.7854	0.7595 n.s.	0.7558 n.s.	0.7556 n.s.	0.7666 n.s.	0.7961 n.s.	0.7544 n.s.
**4/2014**	0.7936	0.7902 n.s.	0.8287 n.s.	0.8405 n.s.	0.8550 n.s.	0.8246 n.s.	0.8347 n.s.

* *p* < 0.05, ** *p* < 0.01, *** *p* < 0.001, n.s.: not significant.

**Table 5:** Some physical and chemical properties of the soil in the experimental site and the incorporated sludge at the beginning of each three-year cycle.

	Harvest Year	Total N (%)	NH_4_ (ppm)	Moisture Content (%)	pH	Organic Matter (%)	CaCO_3_ (%)	Sand (%)	Silt (%)	Clay (%)
**Soil**		0.085	6.2		7.94	1.53	15	39	26	35
**Sewage sludge**	2007	3.6	1102	8.2	6.83	60				
	2009	3.0	980	7.9	7.05	63				
	2010	3.9	1415	9.1	7.90	63				
	2012	4.9	1350	8.5	7.65	62				
